# A look underneath the SiO_2_/4H-SiC interface after N_2_O thermal treatments

**DOI:** 10.3762/bjnano.4.26

**Published:** 2013-04-08

**Authors:** Patrick Fiorenza, Filippo Giannazzo, Lukas K Swanson, Alessia Frazzetto, Simona Lorenti, Mario S Alessandrino, Fabrizio Roccaforte

**Affiliations:** 1 Consiglio Nazionale delle Ricerche - Istituto per la Microelettronica e Microsistemi (CNR-IMM) Strada VIII n. 5, Zona Industriale, 95121, Catania, Italy; 2 Scuola Superiore di Catania - Università degli Studi di Catania, Via Valdisavoia 9, 95123, Catania, Italy; 3 STMicroelectronics, Stadale Primosole 50, 95121, Catania, Italy

**Keywords:** 4H-SiC, metal/oxide/semiconductor, nitrogen incorporation

## Abstract

The electrical compensation effect of the nitrogen incorporation at the SiO_2_/4H-SiC (p-type) interface after thermal treatments in ambient N_2_O is investigated employing both scanning spreading resistance microscopy (SSRM) and scanning capacitance microscopy (SCM). SSRM measurements on p-type 4H-SiC areas selectively exposed to N_2_O at 1150 °C showed an increased resistance compared to the unexposed ones; this indicates the incorporation of electrically active nitrogen-related donors, which compensate the p-type doping in the SiC surface region. Cross-sectional SCM measurements on SiO_2_/4H-SiC metal/oxide/semiconductor (MOS) devices highlighted different active carrier concentration profiles in the first 10 nm underneath the insulator–substrate interface depending on the SiO_2_/4H-SiC roughness.

The electrically active incorporated nitrogen produces both a compensation of the acceptors in the substrate and a reduction of the interface state density (*D*_it_). This result can be correlated with the 4H-SiC surface configuration. In particular, lower *D*_it_ values were obtained for a SiO_2_/SiC interface on faceted SiC than on planar SiC. These effects were explained in terms of the different surface configuration in faceted SiC that enables the simultaneous exposition at the interface of atomic planes with different orientations.

## Introduction

The SiO_2_/4H-SiC interface is the main building block of SiC-based MOSFET devices and its electrostructural quality typically has a direct impact on the device performance in power-electronics applications. In particular, electrically active defects at the SiO_2_/SiC interfaces, such as carbon clusters, silicon suboxide bonds, or intrinsic defects in the near-interfacial oxide layers have been indicated as the origin of the commonly observed low-channel mobility in SiC MOSFETs [1–2]. To alleviate the mobility problem, different postoxidation annealings (POA) of the gate oxide in NO or N_2_O have been explored [3], which can be efficient to provide adequate mobility values in the range of 30–50 cm^2^·V^−1^·s^−1^ [4–5], but still significantly lower than the theoretical limits.

While it is commonly accepted that POA treatments in ambient NO or N_2_O have a beneficial effect on the SiO_2_ insulator and on the SiO_2_/4H-SiC interface due to the passivation of oxide defects and interface traps by the incorporated nitrogen, the impact of these thermal treatments on the electrical properties of the 4H-SiC substrate is still under debate. Recently, Kosugi et al. [6] performed X-ray photoelectron spectroscopy (XPS) measurements on 4H-SiC directly exposed to ambient NO at 1200 °C, demonstrating that a significant density of nitrogen atoms (10^14^ cm^−3^) is incorporated in the 4H-SiC near surface region and remains even after removing the thin (ca. 5 Å) SiO_2_ layer formed on SiC during the annealing by sustained etching in hydrofluoric acid. The same group [7] demonstrated by the electrically detected magnetic resonance technique that shallow donor levels can be associated with a fraction of the incorporated N atoms. However, information on the electrical activation of the incorporated nitrogen and on the depth extension of the nitrogen profile is still lacking. Moreover, the role of the 4H-SiC surface morphology and in particular of the crystallographic planes exposed at the SiO_2_/4H-SiC interface on the nitrogen incorporation have to be clarified.

In this paper, we applied scanning spreading resistance microscopy (SSRM) and scanning capacitance microscopy (SCM) to get a deeper insight into the electrical activation of nitrogen incorporated into 4H-SiC during thermal treatments in N_2_O at 1150 °C. These two scanning probe methods have been applied in the past to study the electrical activation of ion doped and annealed SiC [8–11]. Due to the different measuring principles, i.e., differential capacitance measurements for SCM and current measurements for SSRM, the two techniques exhibit complementary performances especially in terms of dynamic range, with SSRM more suitable for higher concentrations and SCM more sensitive to lower concentrations, respectively. These complementary characters have been fully exploited in the present study. SSRM measurements were performed on p^+^-doped (≈10^19^ cm^−3^) 4H-SiC areas selectively exposed to ambient N_2_O (without the presence of the gate oxide to maximize the effect of nitrogen incorporation), revealing a significant increase of the SiC resistivity with respect to unexposed areas, i.e., a compensation effect from the N-related donors. SCM measurements were performed also on p-doped (≈10^17^ cm^−3^) 4H-SiC exposed to ambient N_2_O through a 30 nm thick gate oxide, to simulate the real metal/oxide/semiconductor stack used in the MOSFET device. To evaluate the impact of 4H-SiC surface morphology on the nitrogen incorporation, SiC samples with properly prepared *flat* or *faceted* surfaces were considered. Cross-sectional SCM measurements provided information on the depth extension of N-compensated 4H-SiC and revealed a more efficient compensation in the faceted 4H-SiC sample.

### Description of the experiment

SSRM is employed to investigate the changes in the resistivity of p^+^-type doped 4H-SiC substrate regions selectively exposed to N_2_O at 1150 °C for four hours, with respect to unexposed ones. The results of this analysis demonstrate that during the low-temperature (1150 °C) thermal treatment, N atoms are incorporated in SiC and a fraction of them occupy a substitutional position, becoming electrically active and acting as donors. The role played by 4H-SiC surface morphology on N incorporation has also been investigated, considering two different samples with a smooth (*flat*) and with a macroscopically stepped (*faceted*) surface, respectively. Details of the surface preparation of the samples can be found in the experimental section. Cross-sectional scanning capacitance microscopy (SCM) was used to profile the active doping concentration in the SiC interfacial region of MOS devices, showing a higher compensation for the *faceted* sample than for the *flat* one. Recently, we demonstrated that MOS on the *faceted* surface exhibit also a lower interface-state density (≈3 × 10^11^ cm^−2^·eV^−1^) with respect to devices on the *flat* surface (≈7 × 10^11^ cm^−2^·eV^−1^) [12–13]. Here, both the different values of *D*_it_ at SiO_2_/4H-SiC interface and the different doping in the near interface SiC region have been explained in terms of the peculiar surface morphology of *faceted* samples, assuming a preferential nitrogen incorporation in the 4H-SiC substrate when it exposes a larger percentile of (11−2*n*) planes.

## Results and Discussion

**Figure 1:**
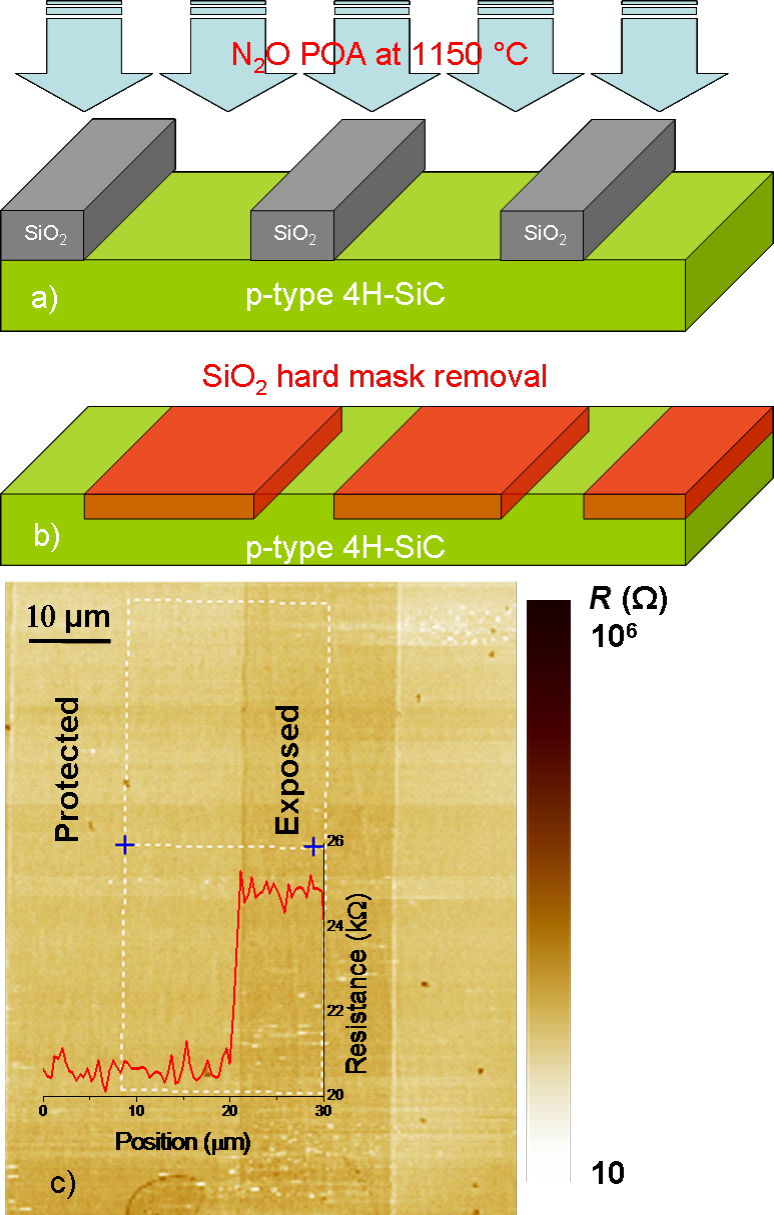
Schematic description of the POA treatment. (a) A SiO_2_ hard mask is used to protect selectively the 4H-SiC during the POA. (b) After removal of the hard mask (by HF wet etch) on the 4H-SiC surface there are regions protected and exposed to the POA. (c) SSRM imaged on the patterned SiC surface; the two dimensional map of the substrate surface. The resistance line profile going from protected to exposed strips demonstrates a higher resistance value in the exposed region.

To gain an insight into the effect of nitrogen on SiC during the annealing in N_2_O, a patterned hard mask was defined onto a p^+^-type SiC surface (as described in the Experimental section) in order to obtain regions selectively exposed and unexposed to N_2_O. After the thermal treatment of the patterned samples (Figure 1a), the hard mask was removed (Figure 1b) followed by SSRM measurements on the bare SiC surface (Figure 1c) [14]. The SSRM map in Figure 1c shows a locally increased resistance in the SiC surface regions that were exposed to N_2_O relative to the regions that were protected by the hard mask. Hence, through SSRM we are able to directly demonstrate that the introduction of nitrogen at the surface of SiC by exposure to N_2_O and the low thermal budget (1150 °C) enables, surprisingly, a compensation effect that reduces the effective concentration of p-type acceptors. Such a scenario has been previously proposed on the basis of physical measurements quantifying nitrogen incorporation and the reduction of *D*_it_ [3]. However, a direct measurement of the local electrical modifications has yet to be reported.

**Figure 2:**
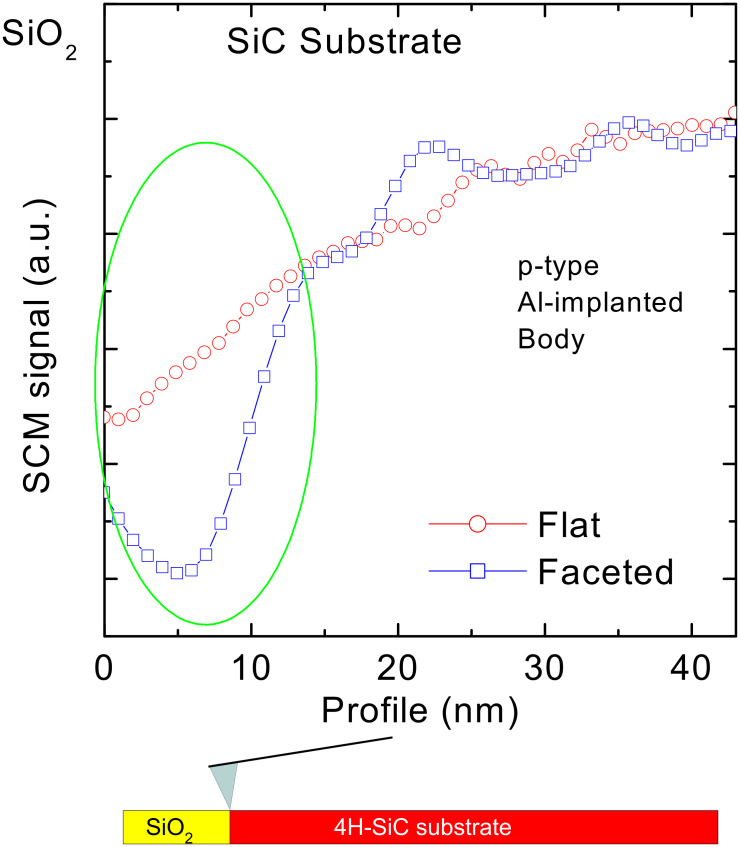
SCM profiling across the SiO_2_/4H-SiC interfaces on the *faceted* (blue squares) and *flat* (red circles) samples respectively.

In the following it will be shown that this compensation effect due to nitrogen incorporation in the SiC near-surface region strongly depends on the SiC morphology, i.e., on the exposed planes at the interface with SiO_2_. High-resolution scanning capacitance microscopy across the interface region was applied to get a deeper insight into this aspect. Figure 2 shows the SCM signal versus depth profiles collected both on the *faceted* and *flat* samples in the 40 nm 4H-SiC region under the SiO_2_/SiC interface. The two depth profiles are laterally averaged over 1 µm. They are almost coincident for depths greater than about 20 nm, while they are significantly different in the SiC interfacial region up to about 15 nm, where the SCM signal in the *faceted* sample is lower than in the *flat* sample. The lower SCM signal indicates a higher compensation of Al acceptors, which is consistent with a more efficient incorporation of substitutional (electrically active) nitrogen atoms in SiC.

The simultaneous presence of different planes at the SiO_2_/4H-SiC interface in the *faceted* sample can be invoked to explain the better nitrogen incorporation during POA, leading to a more efficient passivation of interface traps and to a higher compensation of interfacial SiC. In fact, for a macroscopically stepped surface, the SiO_2_/SiC interface is not only formed on the basal plane (0001), but a significant fraction of the interface area is formed on the (11−2*n*) facets [15]. Accordingly, a different efficiency of nitrogen incorporation on the (0001) and (11−2*n*) facets during the POA process could explain the different *D*_it_ values in the two samples.

**Figure 3:**
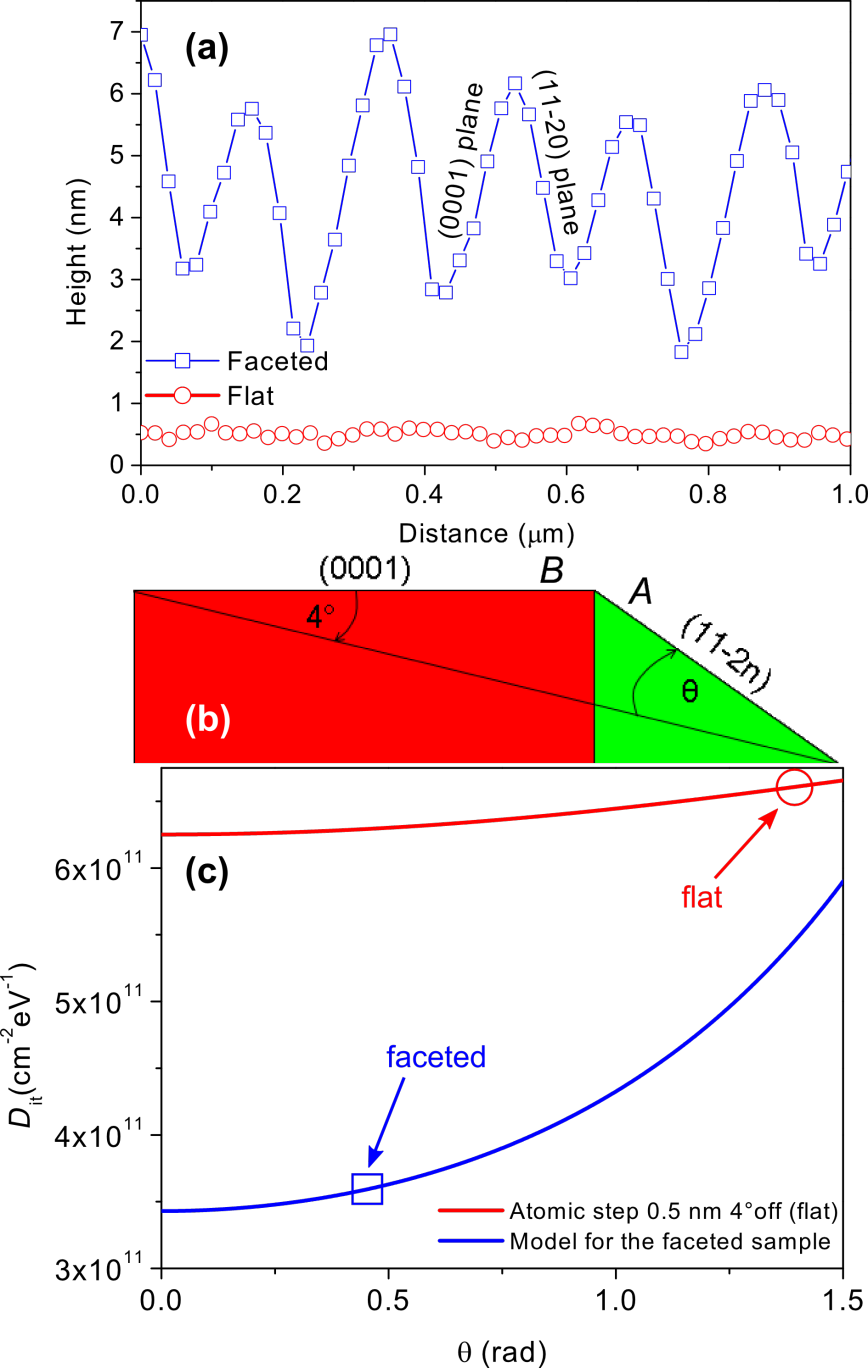
(a) AFM single scan line taken on the surfaces of the *faceted* and *flat* samples. (b) Schematic cross section of the basal-plane–facet orientation considering a 4° off-axis cut angle. (c) Simulated density of interface states for the ideal case and single atomic steps (red), and including the experimental measured facets (AFM section) changing the θ (11−2*n*) facet orientation (blue). The calculated values are compared with the experimental values.

Figure 3a shows the morphology profiles (acquired by AFM data in [12]) of the 4H-SiC substrates surfaces both for the faceted (blue squares) and flat (red circles) samples respectively. The surface roughness (RMS) was 0.36 nm and 1.75 nm for the flat sample and for the faceted sample, respectively. The facets have a typical height of about 5 nm and their orientation is correlated to the original miscut direction of the wafers. In the present case, the substrates are cut along the (0001) basal plane with a 4° off-axis orientation toward the <11−20> direction. Each of these steps exposes both the (0001) basal plane and the plane along the <11−2*n*> direction. On the other hand, the flat sample shows no facets. The line profile in Figure 3a (circles) shows the typical 4H-SiC steps with ca. 0.5 nm height, corresponding to two Si–C pairs. This peculiar morphology of the interface can explain the macroscopic electrical behaviour of the capacitors (*D*_it_).

In fact, for our MOS capacitors fabricated on a faceted surface as in Figure 3a, a significant fraction of the total SiO_2_/4H-SiC interface area is formed on (11−2*n*) planes, with lower *D*_it_ than the basal plane (0001). This latter can justify the reduced *D*_it_ in the faceted sample.

Recently, Saitoh et al. demonstrated [15] the variation in the density of the interface states in MOS capacitors fabricated on 4H-SiC epilayers with different misorientation angles, moving from 8°, i.e., the (0001) largest basal plane, up to 90° toward the <11−20> direction, i.e., the (11−20) plane.

The *D*_it_ at 0.2 eV below the conduction band was found to decrease from *D*_it,(0001);8°-off axis_ = 5 × 10^12^ cm^−2^·eV^−1^ for MOS capacitors fabricated on the (0001) face to *D*_it,(11−20)_ = 3 × 10^11^ cm^−2^·eV^−1^ for capacitors on the (11−20) face. By geometrical calculations based on the AFM morphology in Figure 3a, it was possible to estimate the weighted average between the impact of the (11−2*n*) and the (0001) interface areas in terms of the macroscopic *D*_it_ measured values. The real experimental 4H-SiC structure can be schematically described as in Figure 3b (cross-section configuration).

Considering an ideal *D*_it,(0001)_ = 1 × 10^12^ cm^−2^·eV^−1^ (for the 0° off-axis angle) at 0.2 eV from the conduction band edge, higher than the interface state density in the face (11−20), the total macroscopic density of interface states can be written:

[1]
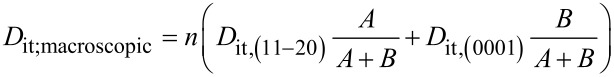


where

[2]
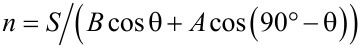


And where *S* is a scale factor, *A* and *B* are the mean experimental dimensions of the (11−2*n*) facets and of the basal planes, respectively (taken from the AFM profiles in Figure 3a). Moreover, the angle θ is the incremental angle with respect to the off-axis cut angle [16].

Equations 1 and 2 predict the total macroscopic *D*_it_, which is the geometrically weighted average between the two limiting conditions, i.e., the interface state density along the (11−20) plane (*D*_it,(11−20)_) and the interface state density along the (0001) plane (*D*_it,(0001)_). For the flat sample the used parameters are based on *A* = 0.5 nm, *B* = *A*/tan 4° (4° miscut angle). The final *D*_it_ value is found taking the value for θ = (90 – 4)° because in the ideal case no facet is formed (Figure 3c). For the faceted sample the used parameters are based on *A* = 4.5 nm and *B* = 5.5 nm. The final *D*_it_ value can be found by taking the variable value θ from 10–20°, because the facet is formed and its angle can vary between 10 and 20°, as reported in the literature [17]. The simulations explain perfectly the experimental results. Figure 3c compares the calculated curves that took to account the geometrical configuration of the SiO_2_/4H-SiC interface in the two cases and the experimental *D*_it_ value measured on MOS prototypes. Recently, J. Rozen et al. [3] pointed out that the *D*_it_ reduction is strongly correlated to the nitrogen incorporation during the POA. Now it is possible to associate the lowering of the density of the interface states (in the faceted sample) with enhanced nitrogen incorporation during the POA when the sample surface exposes a higher percentage of (11−20) planes of the SiO_2_/4H-SiC interface.

## Conclusion

In this paper, the electrical activation of the nitrogen incorporated in the SiC near-surface region during low-temperature POA has been demonstrated. Moreover, nanometre-scale cross-sectional SCM investigation figured out the depth profile of the activated nitrogen. A correlation between the electrostructural properties of SiO_2_/4H-SiC interfaces and the interface-state density of MOS was established. In particular, irrespective of the different interface roughness, lower values of *D*_it_ were found in the faceted sample. The different values of the interface states density can be explained by the peculiar surface morphology of the devices channel region with particular regard to the different nitrogen incorporation through interfaces exposing different ratios between (0001) and (11−20) planes.

## Experimental

Scanning Probe Microscopy (SPM) measurements were carried out by using a Digital Instrument D3100 equipped with the Nanoscope^®^ V controller. Local resistance measurements were carried out by using the scanning spreading resistance module (SSRM) [18–19], and cross-sectional local active-doping profiling was carried out employing differential capacitance (d*C*/d*V*) imaging with the scanning capacitance module (SCM) [20–21].

The MOS capacitor for the measurements of *D*_it_ was fabricated on an n-type 4H-SiC epitaxial layer grown onto heavily doped n^+^-type substrates with a 4° off-axis disorientation towards the <11−20> direction. [22]. A 30 nm thick SiO_2_ layer deposited by plasma-enhanced chemical vapour deposition was used as the gate dielectric. After deposition of the gate oxide, a POA annealing at 1150 °C was performed under an N_2_O atmosphere.

MOSFET devices were fabricated on two different surfaces (*flat* and *faceted*). Both samples were subjected to p-type doping by Al ion implantation and to a subsequent high-temperature (1650 °C) postimplantation annealing for dopant activation. On one sample, the SiC surface was coated by a protective carbon capping layer during the annealing, resulting in a smooth morphology. A rough surface formed by facets exposing both the (0001) basal plane and the (11−2*n*) facets, was obtained for the second sample annealed without the cap layer. MOS capacitors were fabricated on both wafers [12]. Cross-sectional SCM was performed for carrier-depth profiling under the gate oxide region.

The sample for the selective exposure of 4H-SiC bare surface to the postoxidation annealing (POA) was fabricated as follows: the surface of a p^+^-type 4H-SiC layer was selectively exposed to the same POA process in N_2_O, by using a pattern width of 20 μm of exposed substrate separated by 40 µm of protected substrate onto a thick SiO_2_ hard mask. After removing the hard mask, SSRM was performed on bare 4H-SiC exposed to cleaning, wet-etching in diluted HF, and standard rinsing.
